# The Sub-Regional Functional Organization of Neocortical Irritative Epileptic Networks in Pediatric Epilepsy

**DOI:** 10.3389/fneur.2018.00184

**Published:** 2018-03-23

**Authors:** Radek Janca, Pavel Krsek, Petr Jezdik, Roman Cmejla, Martin Tomasek, Vladimir Komarek, Petr Marusic, Premysl Jiruska

**Affiliations:** ^1^Department of Circuit Theory, Faculty of Electrical Engineering, Czech Technical University in Prague, Prague, Czechia; ^2^Department of Pediatric Neurology, 2nd Faculty of Medicine, Charles University, Motol University Hospital, Prague, Czechia; ^3^Department of Neurology, 2nd Faculty of Medicine, Charles University, Motol University Hospital, Prague, Czechia; ^4^Department of Developmental Epileptology, Institute of Physiology, The Czech Academy of Sciences, Prague, Czechia

**Keywords:** interictal epileptiform discharges, brain networks, epilepsy surgery, irritative zone, propagation, neocortical epilepsy

## Abstract

Between seizures, irritative network generates frequent brief synchronous activity, which manifests on the EEG as interictal epileptiform discharges (IEDs). Recent insights into the mechanism of IEDs at the microscopic level have demonstrated a high variance in the recruitment of neuronal populations generating IEDs and a high variability in the trajectories through which IEDs propagate across the brain. These phenomena represent one of the major constraints for precise characterization of network organization and for the utilization of IEDs during presurgical evaluations. We have developed a new approach to dissect human neocortical irritative networks and quantify their properties. We have demonstrated that irritative network has modular nature and it is composed of multiple independent sub-regions, each with specific IED propagation trajectories and differing in the extent of IED activity generated. The global activity of the irritative network is determined by long-term and circadian fluctuations in sub-region spatiotemporal properties. Also, the most active sub-region co-localizes with the seizure onset zone in 12/14 cases. This study demonstrates that principles of recruitment variability and propagation are conserved at the macroscopic level and that they determine irritative network properties in humans. Functional stratification of the irritative network increases the diagnostic yield of intracranial investigations with the potential to improve the outcomes of surgical treatment of neocortical epilepsy.

## Introduction

Focal epilepsy has been traditionally characterized as having its origin in a localized area of one hemisphere. However, experience from surgical treatment has shown that the concept of a restricted focus generating seizures is not accurate for the planning of epilepsy surgery, nor for understanding the pathophysiology of epilepsy. Therefore, the traditional notion of the epileptic focus was replaced by the identification and definition of epileptic zones: the seizure onset zone (SOZ), the irritative zone, the epileptogenic lesion, the functional deficit zone, and the epileptogenic zone, the resection or disconnection of which is necessary for seizure freedom (1, 2). It is well established that resection of the SOZ alone is not always sufficient to achieve seizure freedom and inclusion of the irritative zone, an area that generates interictal epileptiform discharges (IEDs), is necessary. The spatial distribution of the irritative zone is highly variable between patients: in some, it co-localizes with the SOZ (3, 4), while in others, the irritative zone can be extensive, with IEDs distributed over multiple brain areas of one or both hemispheres (5, 6). The resection of the entire irritative zone increases the probability of a favorable outcome after epilepsy surgery (7). However, the entire zone is not equally important in seizure genesis and patients have become seizure free even if a substantial part of the irritative zone remains post-surgery (8). High spatial variability in IED genesis (9), high IED propagation variance (10), and lack of a specific marker of the clinically significant IEDs that delineate the epileptogenic part of the irritative zone are the main constraints limiting the use of IEDs in the planning of surgical resection (11). The low diagnostic value of the irritative zone at present can also be attributed to its clinical definition, which considers this zone as a single entity and does not take into account its network organization. The network approach to epilepsy has already been successfully applied to intracranial recordings, resulting in new insights into the organization of the SOZ, seizure propagation, and mechanisms of ictogenesis (12–14). Unfortunately, network properties underlying the genesis and propagation of IEDs have not been well defined, particularly in patients with neocortical epilepsy.

In the current study, we have examined the functional organization of irritative networks, the long-term dynamics of IED genesis and IED propagation pathways in patients with refractory neocortical epilepsy. For this purpose, we have developed and implemented a novel approach to characterize irritative networks, which is based on recent experimental discoveries about the mechanisms of IEDs (9, 10).

## Materials and Methods

### Clinical Data, Patient, and Data Selection

Fourteen patients (10 female and 4 male) with medically refractory neocortical epilepsy underwent long-term intracranial EEG (iEEG) monitoring as part of their presurgical evaluation. The age of the patients was 9.7 ± 4.0 (median 9.5) years. The average duration of epilepsy was 5.9 ± 3.7 (median 4.5) years. The mean duration of postsurgical follow-up was 5.8 ± 3.2 (median 4.0) years. Seizure outcome was assessed according to Engel’s classification at the last follow-up (15). Demographic and clinical data are summarized in Table 1. Data collection was approved by the institutional ethics committee and informed parental consent was obtained.

**Table 1:** Summary of clinical information of 14 pediatric patients: 10 females, 4 males; age 3–18 years, 9.7 ± 4.0 (median 9.5) in average.

Patient	Epilepsy syndrome	Duration (years)	MR	Etiology (histopathology)	Surgical outcome (Engel sc.)	Follow-up (years)	Type of surgery	Size of surgery	Localization of surgery
P1*	Right SSMA	7	Negative	FCD	I	4	IR	FR	Right SSMA
P2	Left T	9	Positive	Ganglioglima + FCD	I	4	IR	ULR	Basal part of left temporal lobe + AHC
P3	Left F	8	Positive	Encephalitis	I	9	EL	ULR	Left frontal convexity close to SSMA and motor area
P4	Right F	5	Positive	TSC	I	3	IR	FR	Right frontal convexity—prior to motor area
P5	Left SSMA	3	Negative	FCD	I	10	EL	FR	Left SSMA
P6*	Right I	3	Positive	FCD	I	3	IR	FR	Right frontal operculum and insula
P7	Right F	2	Negative	FCD	I	9	IR	ULR	Right frontal convexity—prior to motor area
P8*	Right F	16	Negative	FCD	II	2	IR	ULR	Right orbitofrontal cortex
P9	Left FCT + I	4	Negative	FCD	III	10	IR	FR	Left temporal operculum and dorsal insula
P10*	Right TPO	8	Positive	FCD	IV	4	IR	MLR	Right temporal, parietal and occipital lobe
P11*	Right FT	3	Positive	Encephalitis	IV	3	IR	MLR	Right frontal convexity and temporal lobe
P12	Left T	3	Negative	FCD	IV	10	IR	ULR	Left temporal neocortex
P13	Left CTPO	9	Negative	FCD	IV	11	IR	ULR	Left parietal, occipital lobe, partially temporal lobe
P14	Left FCP	3	Positive	TSC	IV	6	IR	ULR	Left frontal convexity prior to motor cortex

*Sampled recordings by 1 kHz are marked by star*.

*FCD, focal cortical dysplasia; SSMA, supplementary sensorimotor area; TSC, tuberous sclerosis complex; F, frontal; T, temporal; P, parietal; O, occipital; C, central; I, insular; IR, individual resection; EL, extended lesionectomy; FR, focal resection; ULR, unilobar resection; MLR, multilobar resection; AHC, amygdalo-hippocampal complex; MR, magnetic resonance*.

Signals from subdural and/or depth macroelectrodes (Integra, Plainsboro, NJ, USA and Dixi Medical, BESANCON Cedex, France) were amplified (Schwarzer GmbH, Heilbronn, Germany), filtered using anti-aliasing filters at 1/3 of sampling frequency and sampled at a frequency of 200 or 1,000 Hz (Stellate Inc., Montreal, QC, Canada). The 1,000 Hz signals were resampled to 200 Hz by anti-aliasing filters (0.45 of sampling rate). A pair of the implanted electrodes without pathological iEEG was selected as a reference electrode. The iEEG in bipolar channels is a subject of the analysis and the results are presented in relation to these (bipolar) channels unless otherwise stated. Contacts containing large amounts of artifacts were manually removed. On average, 1.0 ± 1.3 (0) contacts per patient were removed. Recordings lasting 91.5 ± 54.6 (median 89.0) min and 72.6 ± 21.5 (65.5) contacts were analyzed for each patient. These data were recorded during both wakefulness and sleep. To examine the long-term dynamics of irritative networks, we analyzed continuous recordings in 8/14 patients with a duration of 57.2 ± 27.2 (median 60.2) h.

### Definition of Epileptic Zones

Brain areas generating IEDs were defined as the irritative zone (2, 6). The SOZ was defined as the area of the brain with the earliest occurrence of ictal discharges (6, 16, 17). Seizure onset times and delineated margins of individual zones were defined and represent an agreement between at least two clinicians who were involved in the presurgical diagnostic work-up. The necessary detailed information about the position of the recording electrodes, locations of individual epileptic zones, and resection margins were provided by clinicians for the purpose of this study. Diagnosis and clinical decisions were made independently of this retrospective study.

### Analysis of Spatial Profile and Distribution of IEDs

**Figure 1:**
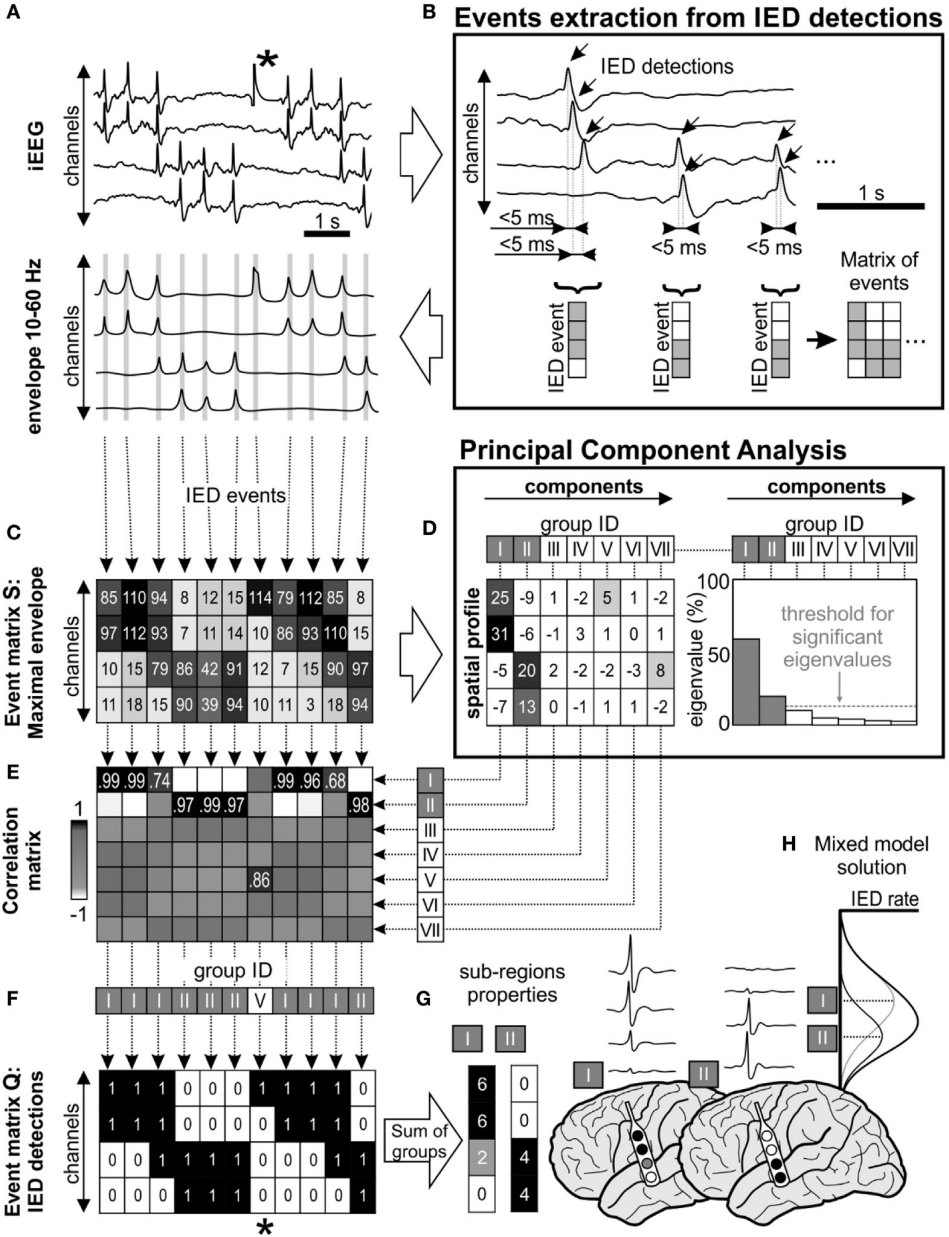
Interictal epileptiform discharge (IED) sorting according to their spatial profile. **(A)** Raw data corresponds with its 10–60 Hz signal envelope, which contains multiple IEDs and also a technical artifact (*). The absolute maximal amplitude and time position of IEDs is defined from the peaks of their envelopes. **(B)** Detected IEDs with a sequential delay of <5 ms are considered as a single IED event (gray lines in the envelope). The events are merged to event time-series matrices. **(C)** Event matrix **S** contains maximum of the envelope amplitude recorded in each channel during the single IED event, **(F)** equally the matrix **Q** stores binary information about detected IEDs (1—IED detected in the channel, 0—no detection). **(D)** Matrix **S** is decomposed using principal component analysis, which extracts components (i.e., common amplitude spatial profiles). Each component is characterized by its eigenvalue, which represents the contribution of columns of matrix **S** before transformation by eigenvectors. In this schematic, first seven principal components were showed (roman numbers I–VII), but only two of them were significant (components I and II) through “random average under permutation” technique (19). This step is followed by sorting detected IED events into groups according to their spatial profile, which combines information from the spatial distribution and amplitude of the envelopes. Each column of matrix **S** (spatial profile of the IED event) is correlated with the all components **(E)** and assigned to the best fitting group **(F)** by the maximal correlation. In this example, detected IEDs were assigned to two significant spatial profile groups (I and II). **(F)** Corresponding columns from matrix **Q** are also assigned to the appropriate group. The spatial profile of each group determines the sub-region. For example, the analysis of the corresponding matrix **Q** provides information about the sub-region’s activity and IED rate **(G)**. The IED profiles were co-registered with fused MRI/computer tomography images, average IED waveform is extracted from raw intracranial EEG (iEEG) for visual inspection. **(H)** The “mixture model” theory allows identification of the existence of local IED sources, which are outside the area of maximal IED occurrence. This area represents joint areas of propagation. Note that the detected artifact (*) was assigned to the non-significant eigenvector in the group V and automatically rejected from subsequent steps of analysis.

The work of Sabolek (10) demonstrated that the propagation trajectories of the early phases of IED propagation were consistent, while trajectories during late phases were divergent and displayed high spatial variance. In other words, the total IED activity of the irritative zone can be represented as a “mixture model” of IED activity generated by multiple sub-populations with variability in propagation trajectories (Figure 1H; Figure S1 in Supplementary Material). Based on these observations, we hypothesized that the spatial distribution of IEDs should differ from a random distribution and consistent spatial IED propagation patterns should be identified within the irritative zone. We, therefore, developed and implemented an IED sorting algorithm that identifies and extracts groups of IEDs through a common spatial profile of the propagation. The algorithm involved the following main steps: detection of single IEDs (18); grouping of propagated IEDs into IED events; identification and extraction of existing spatial profiles of IED events; sorting the IED events according to identified spatial profiles; and quantitative post-processing.

#### IEDs Detection

Interictal epileptiform discharges were detected sequentially through channels from raw iEEG data (18) (Figure 1A). If IEDs were recorded within 5 ms of the previous IED detection in a different channel, the multichannel *IED event* was created (Figure 1B). Each IED event corresponded to one column of time-series *event matrices* and rows represent bipolar channels. The spatial profile of the amplitudes within each IED event is crucial information about the spatial properties of IEDs and their spread across the brain. Therefore, the maximal amplitude of the iEEG (10–60 Hz) signal envelope within events was stored to event matrix **S** (Figure 1C). Additionally, the IED detections were stored to event matrix **Q** as binary information to quantitative evaluation: 1—detected IED, 0—no detection (Figure 1F). The detection time of IEDs in the events was stored to matrix **T** for later time delay estimation of the propagation variability (10). The matrices **Q**, **S**, and **T** had the same dimensionality.

#### Extraction of Spatial Profiles

The time-series event matrices **S** had to be pre-processed to reduce their dimensionality and enhance the stability of the sorting procedure. The high amplitudes artifacts events (columns) and non-epileptic channels (rows) were removed.

Initially, application of Tukey’s criterion to each row of the matrix **S** defined the amplitude range for valid events. Outlier values, which correspond to high amplitude artifact event, were removed from all matrices. Summation of the matrix **Q** in rows defined a total IED rate for each channel, which divided the channels into irritative and non-epileptic activity groups using the k-means algorithm; only irritative channels were utilized for the next step.

Principal component analysis (PCA) was used to extract frequently occurring amplitude spatial profiles of different IED events (20) (see Figures 1C,D). PCA was applied to the pre-processed non-centered matrix **S** to identify principal components (21). The eigenvalues of each principal component defined its contribution to all IED events. The hypothetical presence of a single high eigenvalue suggests a similar amplitude profile between all events, i.e., homogeneous irritative network with a single IED source and propagating pattern. In contrary, irritative networks characterized by the random spatial distribution of IEDs would result in uniform eigenvalues. For real irritative networks and corresponding iEEG data, an information criterion threshold was used to identify and to extract only significant (high eigenvalue) components by “random average under permutation” technique (19). If the eigenvalue was greater than the threshold, it was considered significant (*p* < 0.05), i.e., amplitude spatial profile (component) significantly differed from random spatial distribution. The order of components defined group ID. This step also facilitates removal of events with random spatial distribution (non-epileptiform random transients, noise, etc.).

#### Sorting of IEDs According to Spatial Profile

Each detected event (columns of the matrix **S**) was correlated with all principal components and assigned through maximal Pearson’s correlation coefficient to the best fitting spatial profile (Figure 1E). The sorting procedure resulted in groups of IED events with similar spatial profiles. The event assignment was common to all matrices **S**, **Q**, and **T** (Figure 1F).

After the assignment, waveform averages for each group were generated from raw iEEG and visually validated (Figure 1G). Sub-regions generating non-epileptiform activity were manually removed. Post-processing of the sorted events in matrices generated a final quantitative description of the functional organization and dynamics of the irritative network that are crucial for this study:

(i) *Sub-region* is the area of the irritative network generating a single type of IED event.
(ii) *Sub-region spatial profile* is determined by channels in which single-type IED events are generated and into which they propagate.
(iii) *Sub-region activity* is the percentage of IED events assigned to the sub-region from the total number of IED events. This parameter represents the contribution of the identified neocortical sub-region to global IED activity generated by the whole irritative network.
(iv) *Sub-region IED rate* is defined as the average rate of IEDs in the sub-region. It is determined from the IED rate of each channel within the sub-region per minute.

### Long-Term iEEG Analysis

The IED propagation pathway can be modulated by factors like the level of vigilance, GABAergic inhibition, antiepileptic drugs, and other factors. To examine the spatiotemporal stability of the sub-regions and long-term changes in propagation variance, continuous long-term recordings with an average duration of 57.2 ± 27.2 (60.2) h were available and analyzed in 8/14 patients. The signals were processed by the algorithm, and the results of sorted IED events were divided into 10-min segments. For each segment, we determined the following parameters: average IED rate of the irritative network, sub-region IED rate, activity, and spatial size. The spatial size was defined as number of channels in irritative sub-region part (high IED rate). Temporal profiles of these parameters were compared with a hypnogram. The simplified hypnogram was extracted from channels outside the irritative zone. It was calculated from the average normalized dominant iEEG frequency (half of the zero-crossing number) and energy ratio in time segments in band 1–12 Hz. The hypnogram was validated by visual identification of sleep and wakefulness in video recordings.

### A Multimodality Co-Registration Procedure to Generate Cerebral Maps

Sub-region metrics and other clinical data were incorporated onto cortical maps and co-registered with structural brain images (22). Final multimodal topographic images contain information about electrode placement, sub-region spatial profile, its activity, and IED rate in individual channels. The images are presented with average iEEG waveforms. This approach provides complex information about the properties of each sub-region with a highly precise anatomical localization. The process of visualization is based on rigid co-registration of post-implantation computer tomography and pre-implantation magnetic resonance (MR) images. Co-registration and brain volume extraction were performed using the Statistical Parametric Mapping toolbox (SPM, version 8; The Wellcome Trust Centre for Neuroimaging at UCL, UK, London^1^). Coordinates of contacts were manually extracted in BioImage Suite software (version 3.2; The Yale School of Medicine, New Haven, CT, USA^2^). A custom-made program was developed in MATLAB to fusion and visualize IED sorting results on MR slices or on a 3D brain surface model.

## Results

### Sub-Region Organization of the Irritative Network

Intracranial recordings of 14 patients containing epochs recorded during sleep and wakefulness were analyzed. All the results are presented as a mean ± SD (median). Each dataset contained a mean of 16,868 ± 16,813 (10,071) IED events from which 89.6 ± 11.6 (94.9)% of events were considered significant by PCA, i.e., different from a random distribution. Morphology of the IED events was represented by a spike, polyspike, or spike-and-wave complex. The non-significant events represented false detections due to the presence of artifacts or physiological oscillatory activity (18).

**Figure 2:**
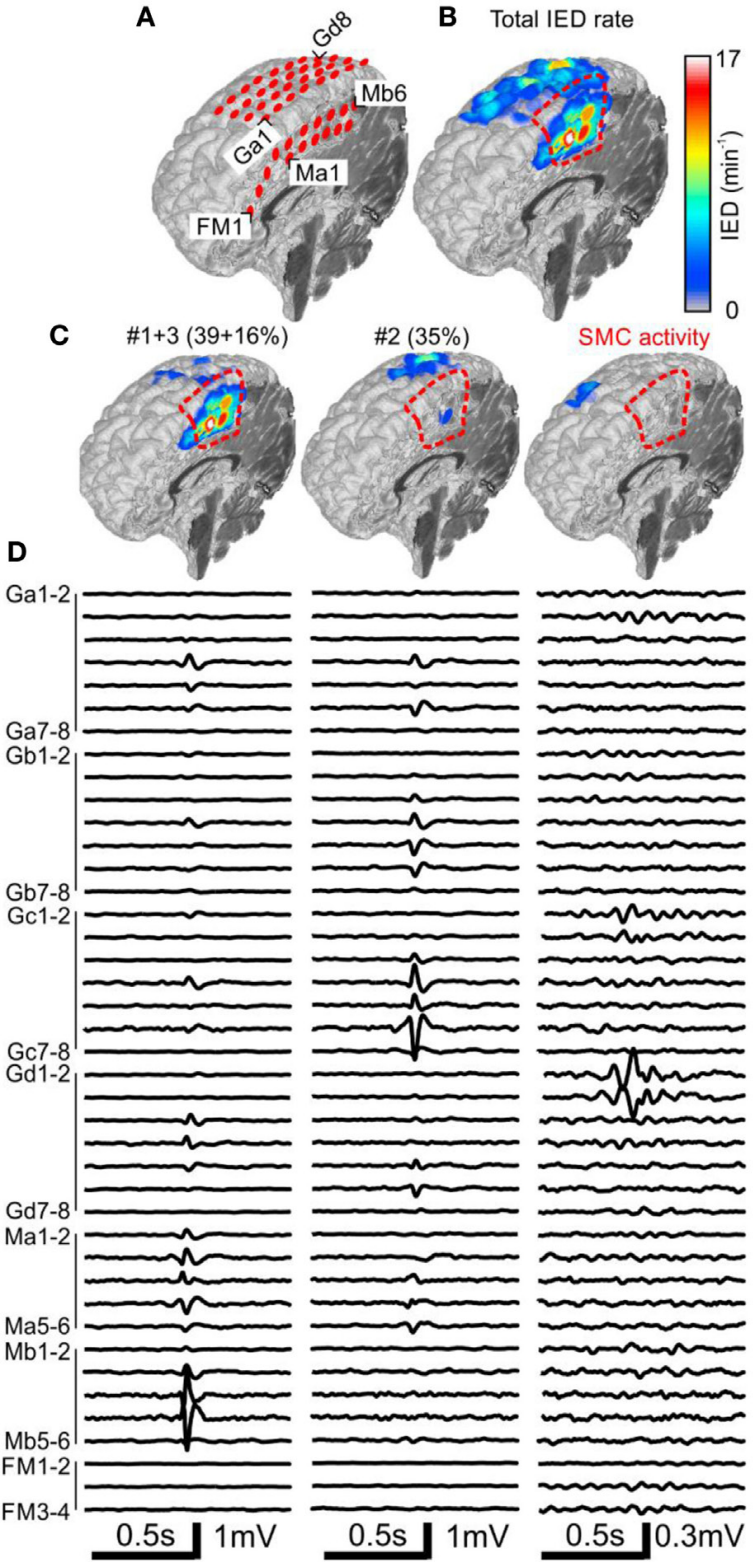
Analysis of the spatial profile of interictal epileptiform discharges (IEDs) in Case 1 (P1). Computer tomography-MRI 3D-reconstruction of the electrode placement is in panel **(A)**. Spatial profile of all detected IEDs shows **(B)**. IED profiles **(C)** and average intracranial EEG waveforms **(D)** of three independent sub-regions identified by the sorting algorithm reveals the heterogeneous nature of the irritative network. The sub-region containing oscillatory activity from the sensorimotor cortex (SMC, el. Gd1–3) was excluded during visual evaluation. Sub-regions #1 and #3 had high spatial overlap and were visualized together. They were localized in the mesial surface under grid M, overlapped with the seizure onset zone (SOZ), and joint sub-region activity was 55%. Sub-region #2 is localized over the convexity (grid G) and its activity was 35%. Resection (dashed red line) involved the SOZ and sub-regions #1 and #3.

**Figure 3:**
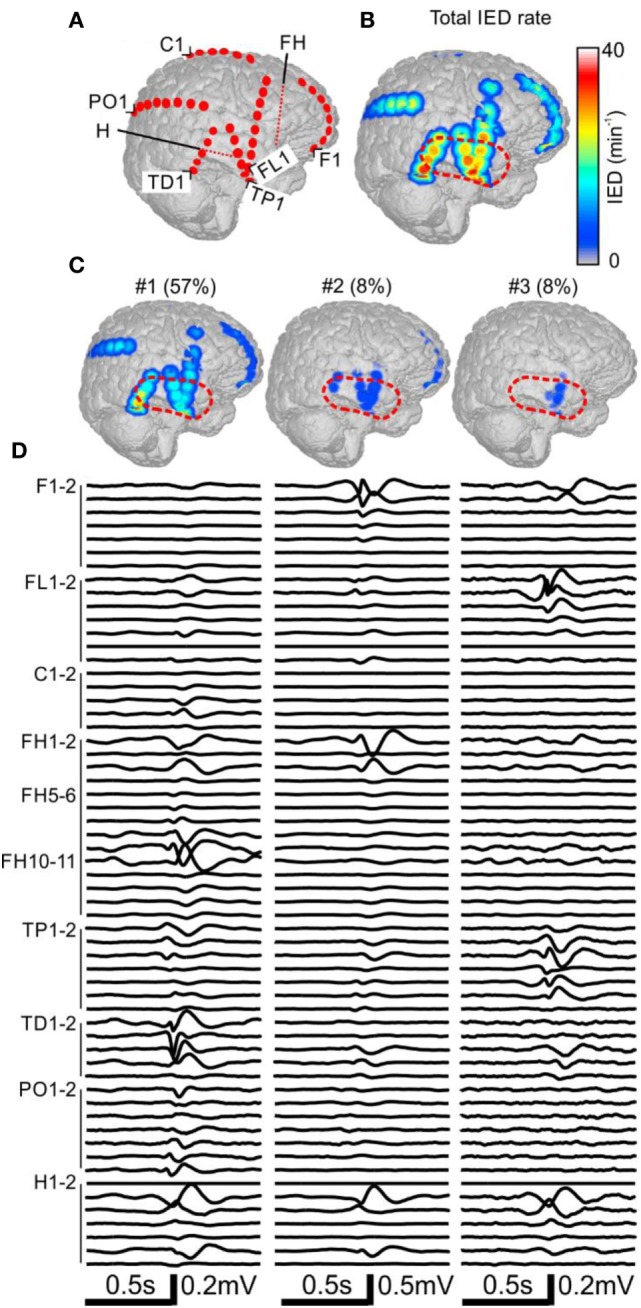
Analysis of the spatial profile of interictal epileptiform discharges (IEDs) in Case 2 (P11). Computer tomography-MRI 3D-reconstruction of the electrode placement is in panel **(A)**. Depth electrodes are visualized schematically and are not visible in surface projections. Spatial profile of all detected IEDs is in panel **(B)**, which were present over large areas of the right hemisphere. Three major identified sub-regions **(C)** and their corresponding activity and average waveforms **(D)** are shown. The most active part of sub-region #1 was not included in the resection (dashed red line).

The major finding of this study is that in each patient, the irritative zone is composed of multiple partly overlapped sub-regions (Figures 2 and 3; Table 2). Each sub-region is composed of channels with a high rate of IEDs (high probability of IED occurrence and propagation) and channels with low IED rates, which correspond to the areas with a low probability of IED propagation (Figures 2 and 3). On average, 11.2 ± 5.5 (11.0) sub-regions per patient were identified. The majority of events (>90%) were initiated in 6.1 ± 3.1 (6.0) sub-regions. The major sub-region generated 45.1 ± 20.3 (38.8)% of IED events. The union of all sub-regions, i.e., the entire irritative network covered an area of 30.4 ± 13.5 (31.5) channels per patient. Meanwhile, the irritative zones marked by clinicians extended over 30.0 ± 15.0 (28.5) electrode contacts. The IED rate of the sub-regions in each patient is shown in Table 2. The major sub-region, i.e., the sub-region with the highest activity, partially overlapped with the SOZ in 12/14 cases and the average proportion of the overlap was 48.7 ± 28.4 (46.5)%. The overlap was calculated as an average of the sensitivity (part of the SOZ) and specificity (part of the sub-region). The complex and modular organization of the irritative network can be well demonstrated in two clinical cases. Results for each patient included in this study are in the Data Sheet S1 in Supplementary Material.

**Table 2:** Summary of the interictal epileptiform discharge (IED) sorting results.

Patient	Number of contacts	Data analyzed (min.)	Number of sub-regions	Average rate of IED events (min^**−**1^)	Max. IED rate in channel (min^**−**1^)	Sub-region’s activities (% of events)
P1*	48	27	5	73	16	39, 35, 16, 8, …
P2	66	104	10	141	18	24, 18, 13, 13, 10, 5, 5, …
P3	65	132	14	324	56	37, 13, 12, 7, 6, 6, 6, 4, …
P4*	73	167	9	216	58	62, 26, 7, …
P5	64	49	8	113	33	38, 31, 14, 9, …
P6	50	50	3	99	22	86, 11, …
P7	98	90	9	30	5	40, 21, 13, 9, 7, …
P8	59	20	2	33	18	77, 23
P9	64	99	17	94	12	25, 19, 14, 7, 6, 6, 3, 3, …
P10	122	60	20	355	49	25, 17, 9, 9, 6, 6,4, 3, 3, …
P11*	67	216	16	256	40	57, 8, 8, 5, 4, 4, 4, …
P12	68	88	12	123	20	29, 26, 22, 9, 3, 3, …
P13	64	59	16	152	36	29, 17, 13, 9, 9, 8, 4, 3, …
P14	108	120	16	170	41	65, 14, 5, 4, 3, 2, …

*Although the maximal IED rate in individual channel does not exceed 58 IED per minute, high events rate signalize independent activity of sub-regions*.

*Sampled recordings by 1 kHz are marked by star*.

### Case 1: Engel I

The patient P1 with refractory epilepsy due to focal cortical dysplasia in the right supplementary sensorimotor area was implanted with subdural electrodes covering the medial and lateral parts of the hemisphere (Figure 2A). The irritative zone was spatially extensive involving all the areas covered by the electrodes (Figure 2B). Visual examination of ictal recordings localized the SOZ to the mesial surface with fast propagation to the lateral channels. Analysis of recorded IEDs revealed the presence of three independent sub-regions (Figures 2C,D). Sub-regions #1 and #3 were visualized together due to a high overlap and generated more than 55% of all IED events. Their location spatially overlapped with the SOZ marked by clinicians. Sub-region #2 was localized laterally, over the convexity, and generated 35% of IED events. Resection involved the SOZ and sub-regions #1 and #3. The patient has been seizure free for 4 years since the surgery. Sensorimotor cortex activity caused false positive IED detection, which were also separated by the algorithm.

### Case 2: Engel IV

The patient P11 with intractable epilepsy caused by encephalitis underwent implantation of subdural and depth electrodes. Visual analysis of invasive data revealed that the SOZ was localized to the right temporal neocortex and that the irritative zone extended over large areas of the right hemisphere. The maximal IED rate was observed in the right temporal neocortex (Figure 3). Application of sorting analyses to intracranial data identified 16 sub-regions. Sub-region #1 generated 57% of all IED events and its spatial distribution involved multiple areas in the temporal, frontal, and occipital lobes with the maximum in the laterobasal temporo-occipital neocortex next to the SOZ (Figures 3C,D). Sub-region #2 caused 8% of events and was localized to the right temporal pole. Sub-region #3 was localized to the basal temporal neocortex and generated 8% of events. The activity of the remaining sub-regions was less than 6% and they were distributed over various parts of the right hemisphere: hippocampus, parietal lobe, basal part of the frontal lobe, dorsal part of the temporal lobe, and convexity of the frontal lobe. The patient underwent a large right temporal lobe resection, which involved the SOZ and sub-regions #2 and #3. The most active part of sub-region #1 was not included in the resection. Seizures recurred 9 months after the surgery (Engel class IV).

### Time Versus Spatial Propagation Profile

**Figure 4:**
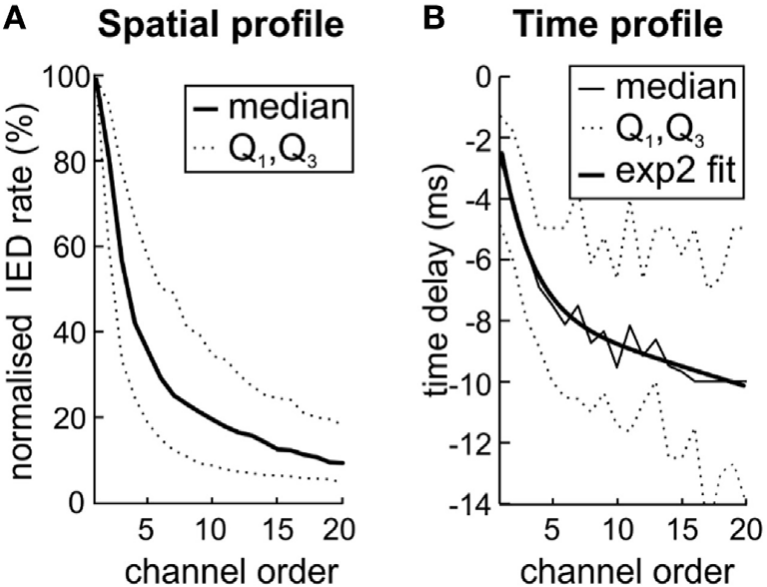
The variation in interictal epileptiform discharge (IED) propagation through pathways (10) causes uncertainties of spike time tracking to source. **(A)** The decreasing IED rate trend reflects the IED amplitude loss during propagation from local source to farther areas by variable pathways. **(B)** Channels with the highest rate (low rank) also display the lowest time delay. It suggests that high IED rate together with low time delay mark the site of origin of IEDs within the sub-region and also the areas with high probability of IED propagation. The two-term exponential model was used for curve fitting; the equation for the model is the following *f*(*x*) = *a* exp(*bx*) + *c* exp(*dx*). Q1 and Q3 mark quartiles.

Next, we have examined whether the spatial distribution of sub-regions IED rate reflects the propagation pathway of IED events. Based on Sabolek’s work (10), we explored if the areas with high IED rate correspond to the areas of IED origin and areas with early and reliable IED propagation. By contrast, we tested if areas with high variability of the IED propagation will be distant from the site of IED origin, displaying large time delay and low IED rate. We compared the sub-region IED rate with the average time delay within IED events. For each sub-region (*N* = 205), the channels were ordered according to the IED rate, from the highest to the lowest value. IED rates in each channel were normalized to the interval from 0 to 100% according to the maximal IED rate observed within the sub-region. Figure 4A shows the average of all sub-regions. The results demonstrated a steep decrease of IED rate from the IED site of origin to the area of propagation. An analogous relationship was observed for time delay (Figure 4B). Channels with the lowest IED rate (i.e., high rank) displayed the largest time delay [Spearman correlation; 0.58 ± 0.29 (median 0.57)]. It seems that spatial distribution of IED rate within each sub-region reflect preferred propagation pathways of IED event.

### Long-Term Dynamics of Sub-Region Properties

**Figure 5:**
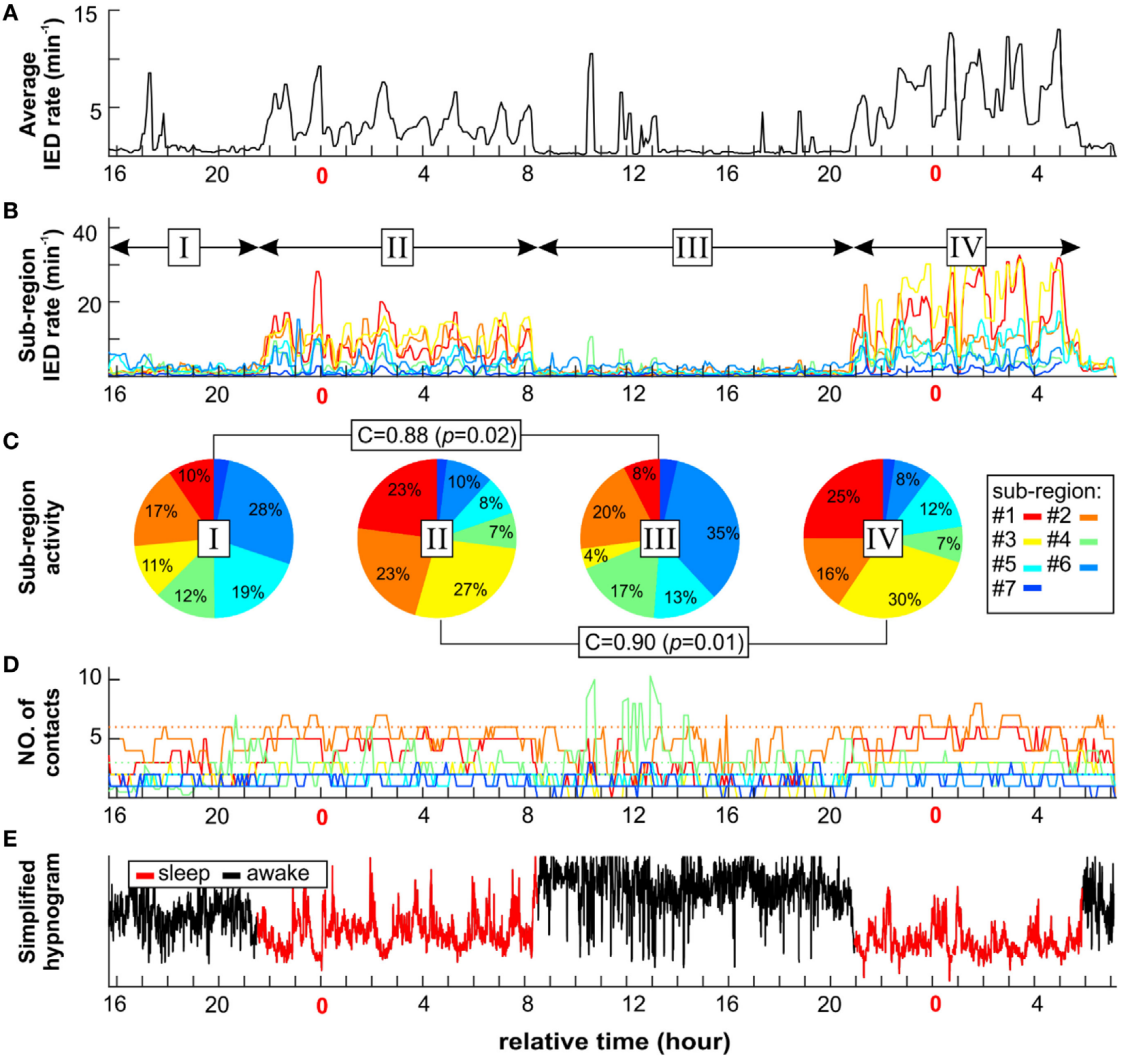
An example of long-term dynamics (circadian and ultradian) of the irritative network in patient P2 demonstrates a non-uniform effect of vigilance to sub-regions interictal activity. Sleep is characterized by increased irritative network interictal epileptiform discharge (IED) rate **(A)**. On the sub-regional level, long-term dynamics of each sub-region display various temporal profiles of IED rate **(B)** and spatial size **(D)**. Each sub-region contribution to total IED rate varies depending mainly on vigilance state **(C)**. However, the correlation between awake stages activity ratio (I and III) and sleep stages (II and IV) indicates two different network settings. **(E)** Corresponding simplified hypnogram shows sleep marked by red color.

**Figure 6:**
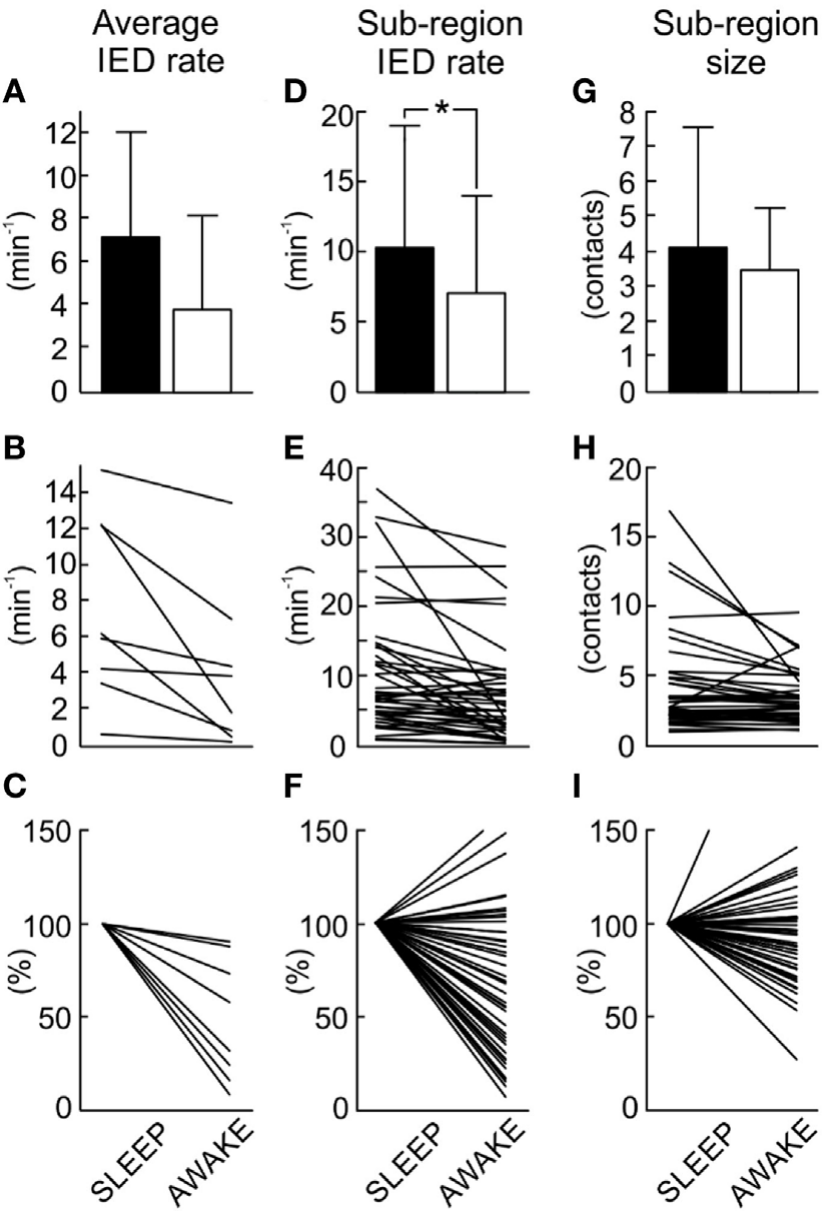
Quantification and comparison of sub-region dynamics during sleep and wakefulness. The average interictal epileptiform discharge (IED) rate of the entire irritative network **(A)** decreases during wakefulness. The average irritative network IED rate in each patient **(B)** shows a significant decrease of the IED rate in all patients (Mann–Whitney test, *p* < 0.001). Normalized network IED rate **(C)** displays proportional changes when compared to sleep values. Group data of sub-region IED rate **(D)** demonstrates a decrease in the rate during wakefulness. Sleep versus wakefulness changes in sub-region IED rate **(E)** demonstrates the presence of both: an increase and decrease in IED rate. In 83.7% of sub-regions, these changes were significant at *p* < 0.05. Normalized changes of sub-region IED rate shows **(F)**. Wakefulness is usually associated with the reduction of the spatial extent of the sub-regions **(G)**. Changes in spatial extent of each sub-region **(H)** and their normalized values **(I)** are shown. In 69.8% of cases, these changes were significant at *p* < 0.05. The bar marks mean value, “whiskers” SD. The asterisk marks statistical significance at *p* < 0.05.

In next step, we have explored whether number of sub-regions, their properties, and IED propagation remain stable over long term. In each patient with available long-term iEEG recordings (8/14), the quantitative parameters of the irritative network and sub-region properties displayed considerate variability (Figures 5 and 6). The average number of identified IED events was 426,565 ± 332,126 (454,405). During wakefulness, the IED rate of the irritative network was significantly lower for all tested patients (Mann–Whitney test, *p* < 0.001; Figure 6B). It dropped to 48.2 ± 33.1 (44.2)% of the IED rate values observed during sleep (Figure 6C). Statistically significant circadian changes of IED rates were observed in 83.7% of sub-regions (Mann–Whitney test, *p* < 0.05). In 76.7% of sub-regions, activity decreased to 52.3 ± 27.9 (54.7)% of sleep values (Figures 6E,F). By contrast, 23.3% of sub-regions increased their activity to 119 ± 21.3 (111.0)% of sleep values (Figure 6F). Next, we examined the impact of circadian rhythms on the spatial profile of individual sub-regions. None of the sub-regions demonstrated a shift in their position. Only shrinkage or expansion of their spatial profile occurred. During wakefulness, 62.8% of sub-regions decreased their spatial extent to 77.7 ± 16.4 (81.3)%, while 37.2% of sub-regions increased their spatial distribution (Figures 6H,I).

## Discussion

### Functional Organization of the Irritative Network

In this study, we have identified and described new features of the functional organization of the neocortical irritative network, which have important implications for the pathophysiology of neocortical epilepsy and for the improvement of epilepsy surgery planning. Numerous clinical studies have aimed to functionally stratify the irritative network according to various parameters of IEDs (high rate, high amplitude, and origin), to help localize the SOZ and tailor resections (3, 8, 23). However, these studies did not appreciate the organizational complexity of the irritative network. The main finding is that the irritative zone has a heterogeneous and modular nature due to the existence of multiple sub-regions that can be reliably identified, separated, their properties quantified, and their relationship with other zones determined.

Heterogeneity within irritative networks has been documented in temporal lobe epilepsy (23, 24). In this type of epilepsy, IEDs and seizures are generated by different limbic structures and propagate through various pathways of the limbic network (25, 26). Bourien et al. (24) showed that IEDs in temporal lobe epilepsy can be generated by subsets of limbic structures and identification of these subsets can provide information to discriminate between mesial or lateral temporal lobe epilepsy. In the neocortex, this type of irritative network organization is not so well established, but the results of the present study suggest that the concept of a multi-site origin of IEDs and sub-region organization can be equally important. Each sub-region represents a part of the network that is capable of generating synchronous neuronal activation, which manifests on the EEG as an IED event. This observation provides intracranial electrophysiological evidence for the results obtained from dipole modeling studies, which have identified the presence of multiple sources of interictal activity in patients with epilepsy of neocortical origin (27, 28). Also, several EEG-fMRI studies have demonstrated that neocortical IEDs can be accompanied by changes in the blood-oxygen-level dependent signal in multiple, independent areas of the neocortex, indicating the activation of distinct neuronal populations (6, 29–33). Khoo et al. (34) showed that the irritative zone was composed of multiple areas of activation, but the region with the most significant hemodynamic response to an IED delineated the subset of the irritative zone that generated the seizures. We can hypothesize that this fMRI feature could reflect the activity generated by the most active sub-region identified in intracranial recordings, which displays spatial overlap with the SOZ.

Such a complex nature of the irritative network may emerge from its network structure, but it can also be attributed to the underlying structural alteration of connectivity associated with specific epileptogenic lesions (35). In particular, developmental cortical abnormalities (the main pathology identified in this study) can be more spatially extensive with very complex connectivity patterns (6, 36), resulting in large areas of the brain having the capacity to generate and propagate IEDs. In these cases, the exact identification of the critical components of the network can be a challenging task due to the presence of multiple local and remote areas of epileptogenicity, and it often requires invasive exploration and a detailed understanding of the irritative network (1, 37). In these clinical scenarios, our novel approach for analysis of the irritative zone substantially increases the information yield of intracranial recordings. The results provide clinicians with unbiased and quantitative information about the detailed structure of the irritative zone, which can substantially facilitate the interpretation of intracranial recordings, the decision process, and resection planning. Whether irritative networks in patients with neocortical epilepsy due to more localized epileptogenic lesions (neoplasms and vascular malformations) possess such complex organization remains unknown. In such cases, results of the presurgical examination are often convergent, and implantation of intracranial electrodes is not necessary. Further studies are essential to precisely determine the diagnostic potential of the functional stratification, taking into account factors, such as age, epilepsy duration, lesion type, and its localization. Also, spatial sampling error due to incomplete coverage of the brain could be an additional factor to consider when interpreting results from this type of study.

### Pathophysiological Implications and IED Propagations

The whole analytical approach to the stratification of the irritative zone was designed according to current knowledge of the principles of IED generation and their propagation through neural tissue. A study by Sabolek (10) showed that as an IED propagates along the cortex, the pathway variability increases and later phases of the propagated discharge can follow different pathway trajectories. The main determinants of propagation variability were GABAergic inhibition and activity of interneuronal networks (9, 38, 39). In our study, we demonstrated that similar principles can be observed on macroscopic scales in humans. The existence of multiple sub-regions confirms the involvement of multiple independent neuronal populations in IED genesis, while long-term changes in the spatial profiles of the sub-regions reflect the variance in IED propagation pathways. Each sub-region is composed of areas with a high IED rate and a reliable occurrence of IEDs and areas with low reliability of IED occurrence. It is considered that areas of low reliability represent pathways with high propagation variance over time.

In addition, we demonstrate that IED genesis and propagation pathways are substantially modified by sleep and wakefulness, which influence long-term fluctuations in the sub-region’s spatial profile (i.e., propagation). Sleep is well known as modulator of epileptiform activity, affecting parameters such as rate of IED, high-frequency oscillations, or the probability of seizure occurrence (40–42). In particular, NREM sleep is a state associated with an increased propensity to synchronize brain activity and its facilitating effect on the genesis of IEDs and their propagation are well documented in temporal lobe epilepsy (43, 44). We demonstrate that sleep/wakefulness can dramatically change the activity of sub-regions. While specific sub-regions can be active during sleep, the dominance in activity can shift to different sub-regions during wakefulness. The capability to capture such changes in irritative network dynamics is also essential for correct interpretation of intracranial recordings. For diagnostic implications, it is important that sleep does not influence the number and location of sub-regions, but the proportion of IED activity between sub-regions may change.

## Ethics Statement

Data collection was approved by the institutional ethics committee of the Motol University Hospital and informed parental consent was obtained.

## Author Contributions

RJ, PK, PJi, and PM conceived of the presented idea. RJ and PJi wrote the manuscript with support from PM and PK. RJ, PJe, and RC developed the theory and performed the analytic calculations. PK, MT, PM, and VK collected the patient data, performed the medical examination and clinical results. PJi, PM, RC, and VK helped supervise the project.

## Conflict of Interest Statement

The authors declare that the research was conducted in the absence of any commercial or financial relationships that could be construed as a potential conflict of interest.

## Footnotes

^1^www.fil.ion.ucl.ac.uk/spm (accessed March 14, 2018).

^2^http://bioimagesuite.yale.edu (accessed March 14, 2018).
