# Corrigendum: The Sub-Regional Functional Organization of Neocortical Irritative Epileptic Networks in Pediatric Epilepsy

**DOI:** 10.3389/fneur.2019.00631

**Published:** 2019-06-14

**Authors:** Radek Janca, Pavel Krsek, Petr Jezdik, Roman Cmejla, Martin Tomasek, Vladimir Komarek, Petr Marusic, Premysl Jiruska

**Affiliations:** ^1^Department of Circuit Theory, Faculty of Electrical Engineering, Czech Technical University in Prague, Prague, Czechia; ^2^Department of Pediatric Neurology, 2nd Faculty of Medicine, Charles University, Motol University Hospital, Prague, Czechia; ^3^Department of Neurology, 2nd Faculty of Medicine, Charles University, Motol University Hospital, Prague, Czechia; ^4^Department of Developmental Epileptology, Institute of Physiology, The Czech Academy of Sciences, Prague, Czechia

**Keywords:** interictal epileptiform discharges, brain networks, epilepsy surgery, irritative zone, propagation, neocortical epilepsy

In the original article, there was an error. In the Acknowledgements statement, we referenced funding that is not relevant to this article.

A correction has been made to the **Acknowledgements** section:

“We would like to thank Prof. John G. R. Jefferys and Dr. Jaroslav Hlinka for their valuable comments. This manuscript was supported exclusively by a grant from the Ministry of Health of the Czech Republic (AZV 15-29835A).”

The authors apologize for this error and state that this does not change the scientific conclusions of the article in any way. The original article has been updated.

