# Supplementary material for: The Sub-Regional Functional Organization of Neocortical Irritative Epileptic Networks in Pediatric Epilepsy
Source: Front Neurol. 2018 Mar 23;9:184. doi: 10.3389/fneur.2018.00184 (PMC5876241; doi:10.3389/fneur.2018.00184)
Supplement: Supplementary file 1 [file data_sheet_1.PDF]

Supplementary Material

The sub-regional functional organization of neocortical irritative epileptic networks in pediatric epilepsy

Radek Janca, Pavel Krsek, Petr Jezdik, Roman Cmejla, Martin Tomasek, Vladimir Komarek, Petr Marusic, Premysl Jiruska

*** Correspondence:** Radek Janca: jancarad@fel.cvut.cz

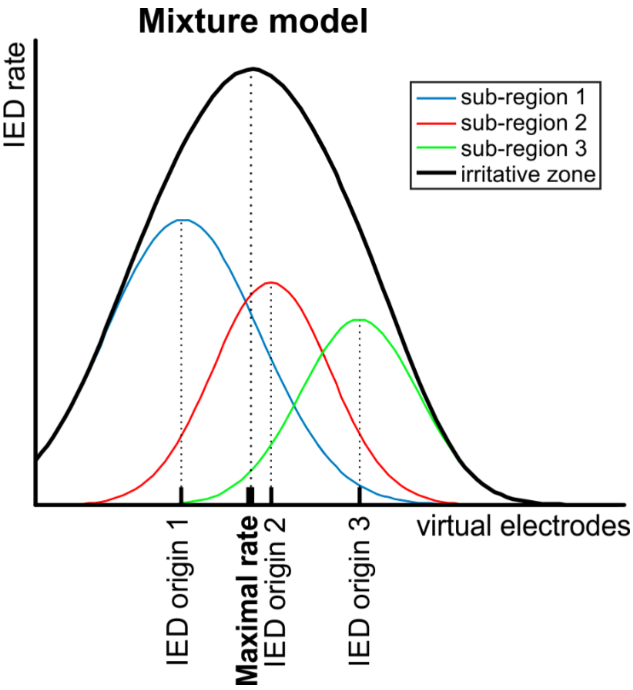

Supplementary Figure 1. The mixture model of the IED spatial distribution. The irritative zone composed from sub-regions with independent IED generators can result in the formation of a region with maximal IED rate. In the visual assessment of intracranial recordings, such region can be incorrectly interpreted as a highly epileptogenic region. Using the knowledge about the spatial propagation profile of IED, the developed algorithm allows to stratify the organization of the irritative zone and identify and separate the existence of sub-region generating IED activity. Information about the detailed structure of irritative zone provides highly relevant clinical information for resection planning.

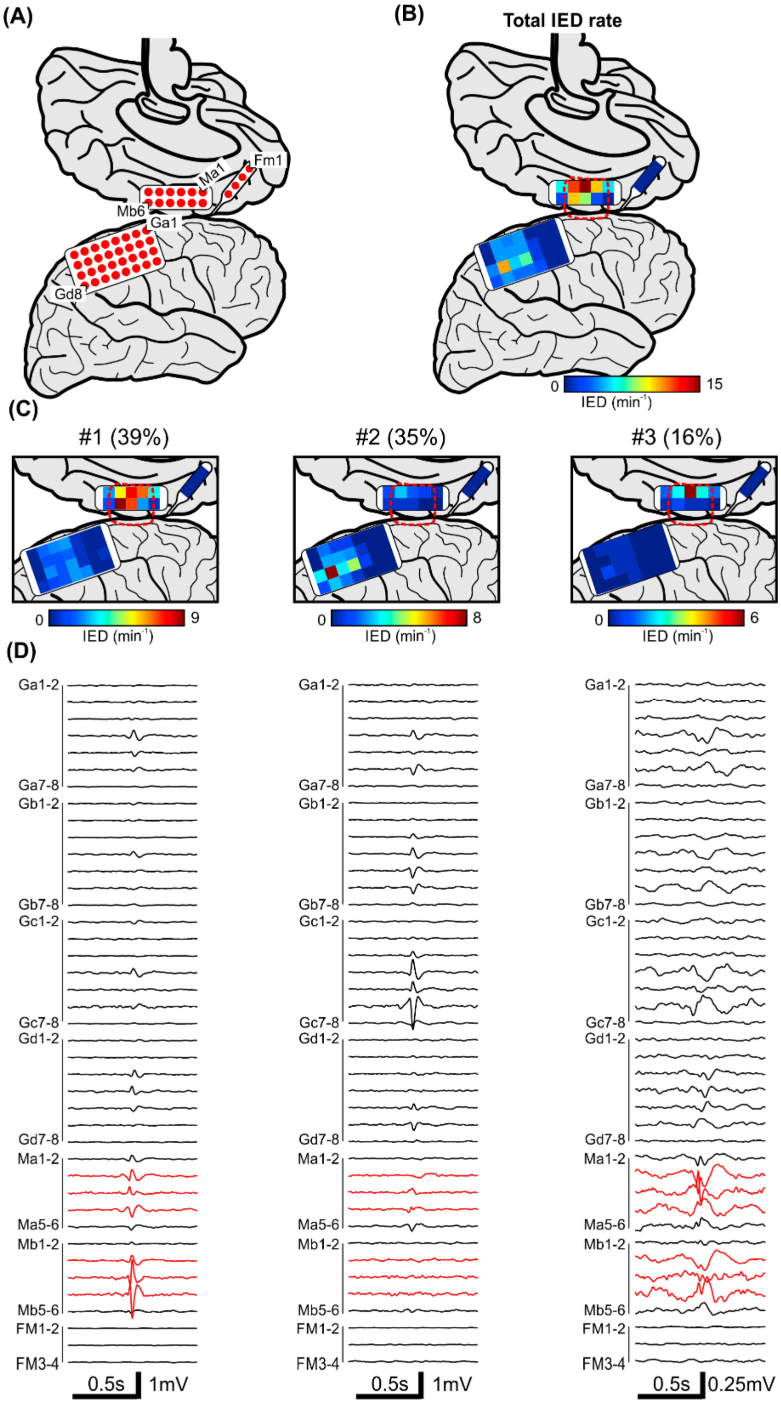

Supplementary Figure 2. Analysis of the spatial profile of IEDs (patient P1). (A) The schematics of electrode location. (B) Spatial profile of all detected IEDs. (C) Three identified sub-regions and their corresponding activity and average waveforms (D) are shown. The most active sub-region #1 and sub-region 3# were included in the resection (red line). The patient's outcome is Engel I.

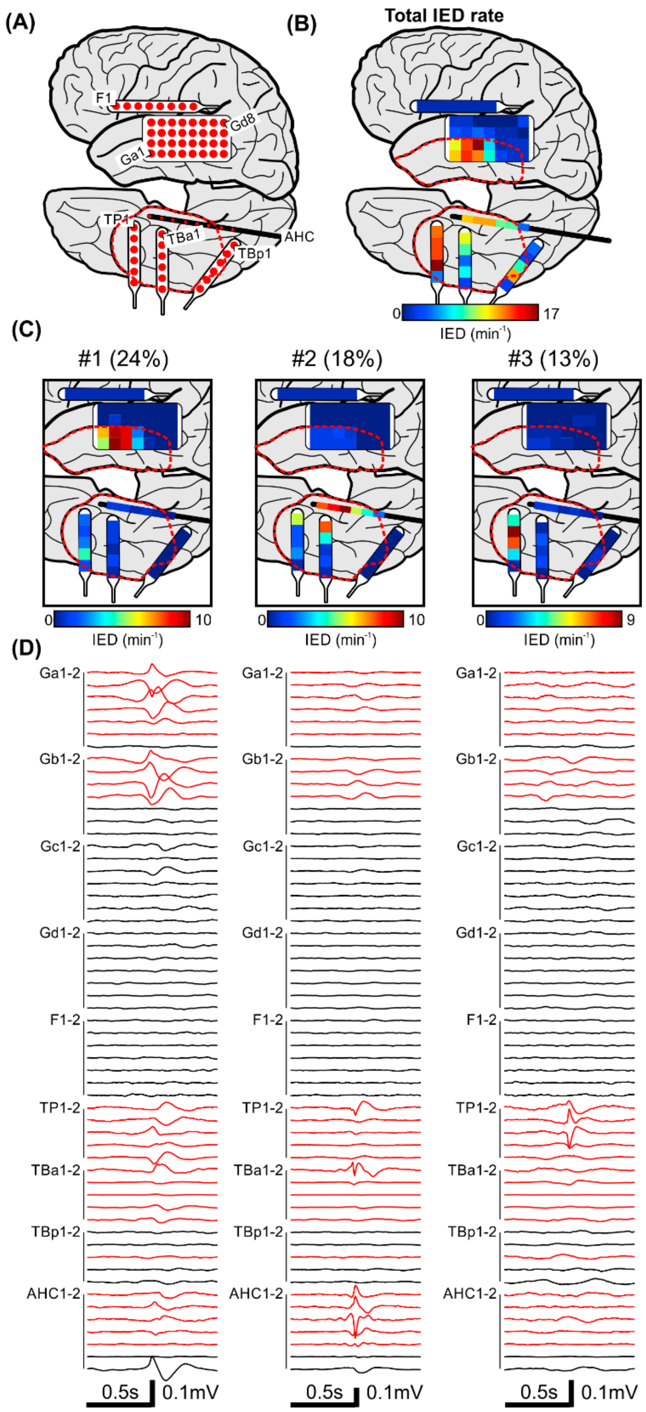

Supplementary Figure 3. Analysis of the spatial profile of IEDs (patient P2). (A) The schematics of electrode placement. (B) Spatial profile of all detected IEDs. (C) Spatial profile of the three most active sub-regions and their corresponding activity. (D) Average waveforms for each sub-region. These sub-regions were included in the resection (red line). The patient's outcome is Engel I.

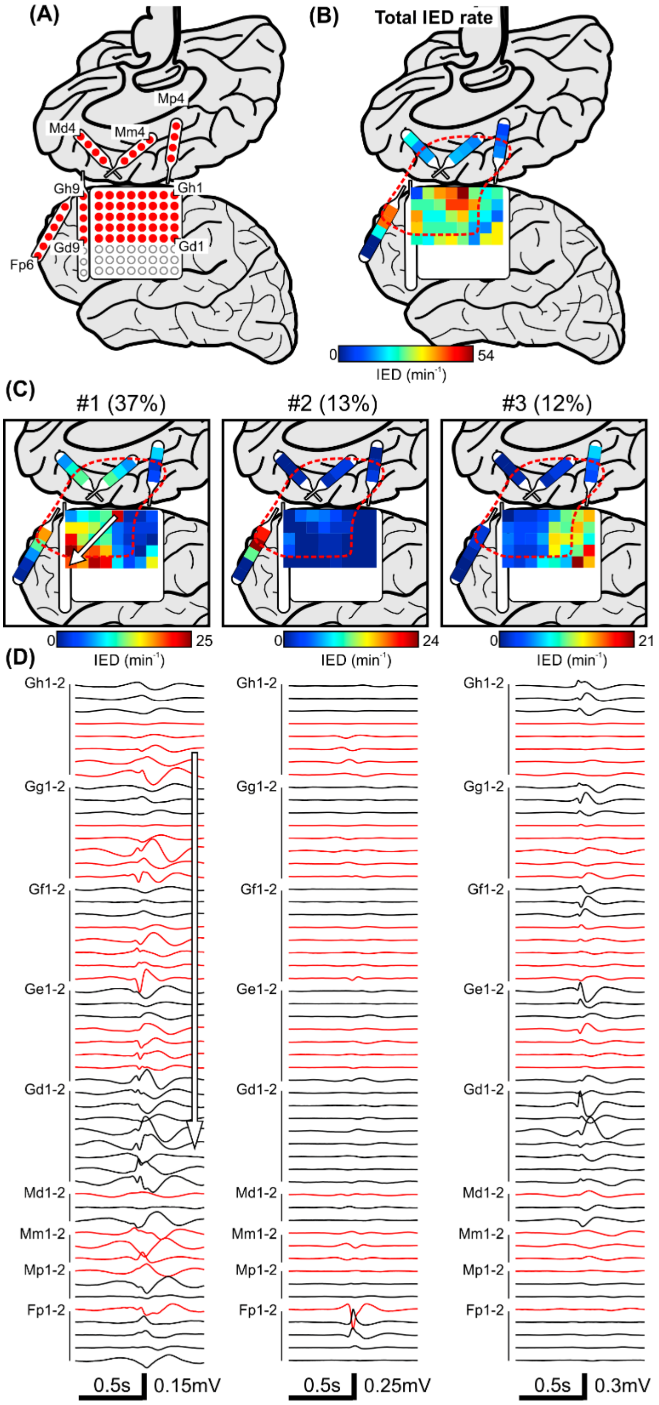

Supplementary Figure 4. Analysis of the spatial profile of IEDs (patient P3). (A) The schematics of electrode placement. (B) Spatial profile of all detected IEDs. (C)Three main identified sub-regions, their corresponding activity and average waveforms (D). The majority part of sub-region #1 was included to resection (red line). The edge of the resection in electrodes Gd3-8 represents the area of late propagation from Gh4-5 (the arrow). The patient's outcome is Engel I.

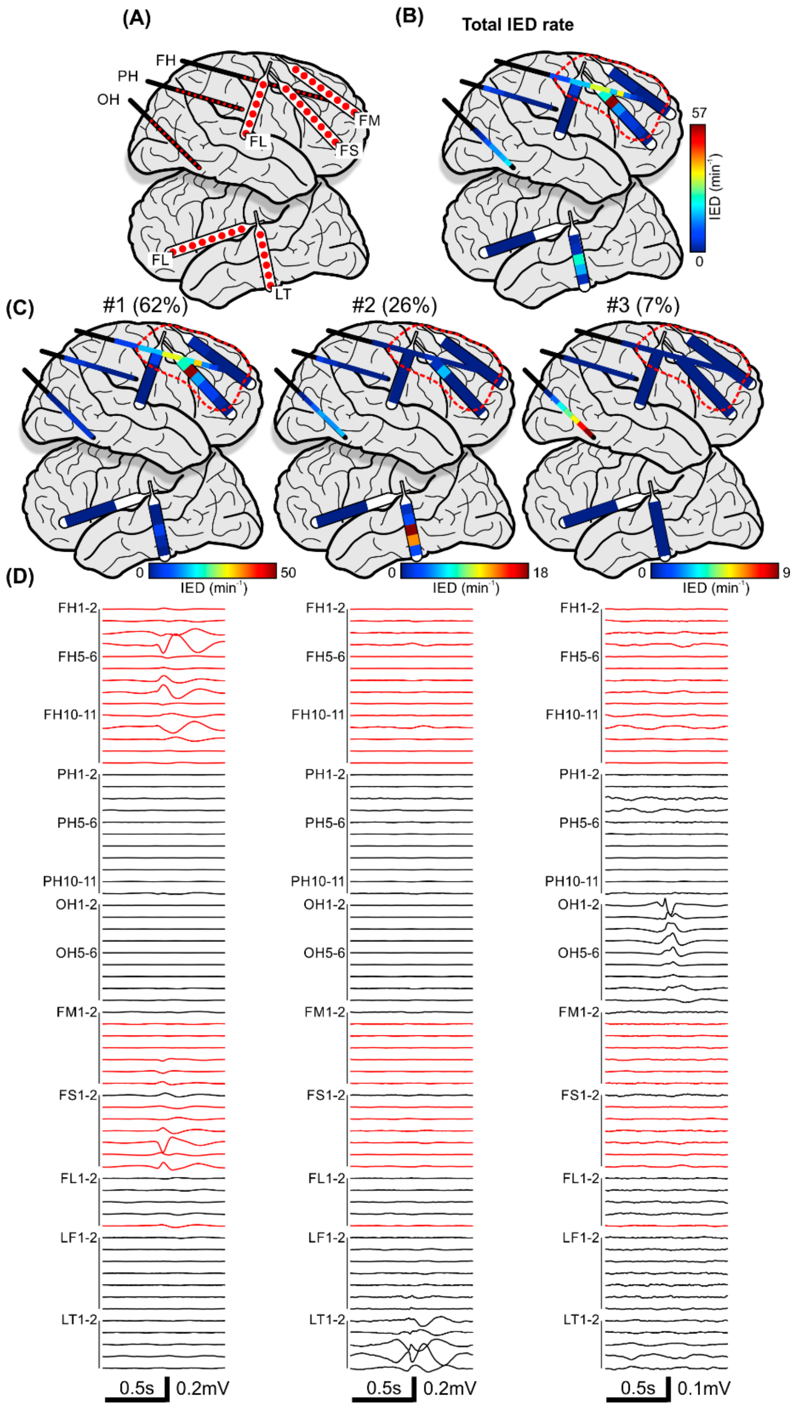

Supplementary Figure 5. Analysis of the spatial profile of IEDs (patient P4). (A)The schematics of electrode placement is schematically in. (B) Spatial profile of all detected IEDs. (C) Three identified sub-regions, their corresponding activity and average waveforms (D). The sub-region #1 co-localized with a tuber in the frontal lobe and it was included in resection (red line). Other sub-regions correspond to other lesions localized to lateral temporal lobe and occipital area. The patient's outcome is Engel I.

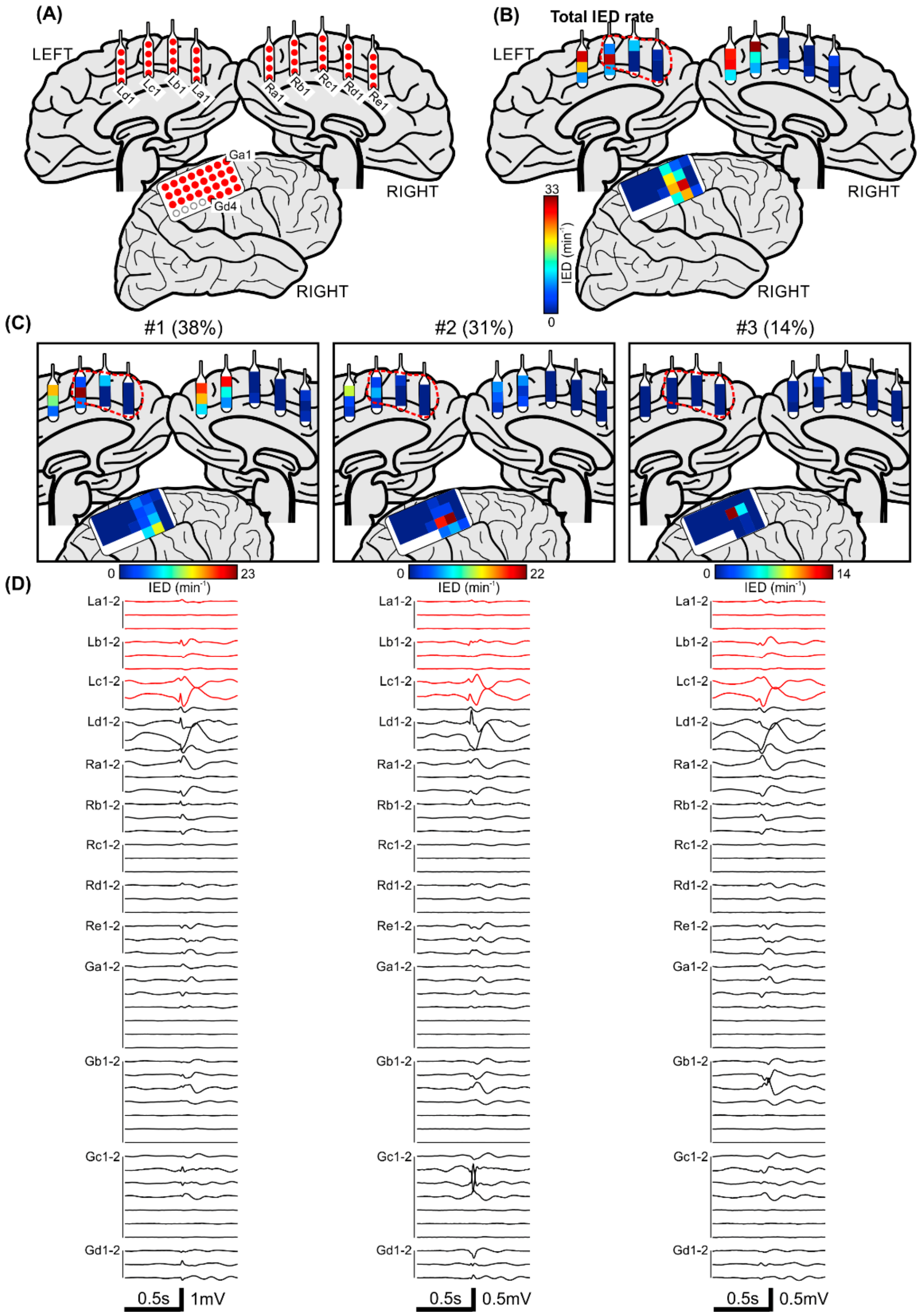

Supplementary Figure 6. Analysis of the spatial profile of IEDs (patient P5). (A) The position of electrodes. (B) Spatial profile of all detected IEDs distributed over frontal lobe area bilaterally. (C) Three major sub-regions and their corresponding activity and average IED waveforms (D). Although the maximum IEDs were in the right mesial frontal area, the most active sub-region #1 was located in the left hemisphere, co-localized with SOZ and in resection (red line). The patient's outcome is Engel I.

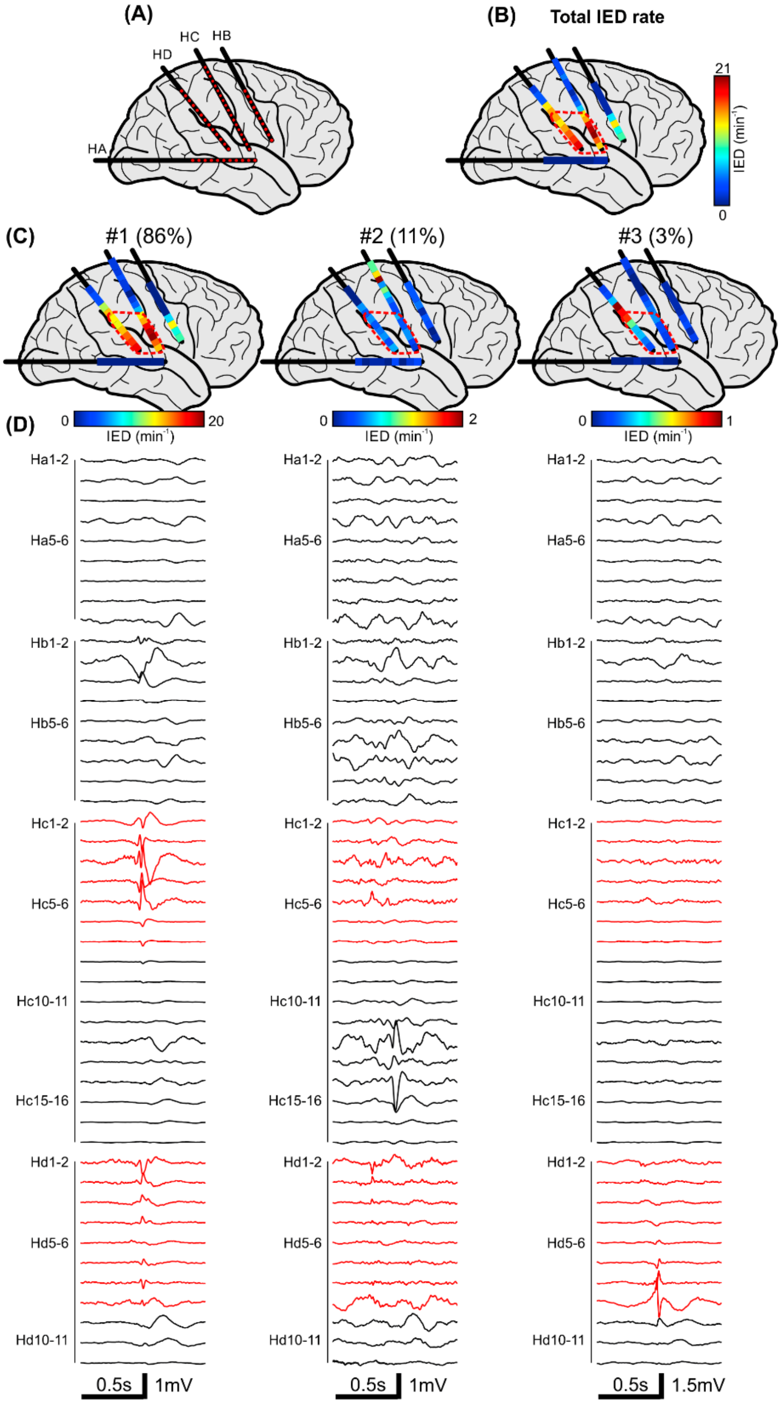

Supplementary Figure 7. Analysis of the spatial profile of IEDs (patient P6). (A) Schematics of electrode placement. (B) Spatial profile of all detected IEDs. (C) Main sub-regions and corresponding average IED waveforms (D). The most active sub-region #1 and minor sub-region #3 were located in the insular cortex, and they were included in resection (red lines). The remaining minor sub-region #2 was localized in the sensorimotor cortex. The patient's outcome is Engel I.

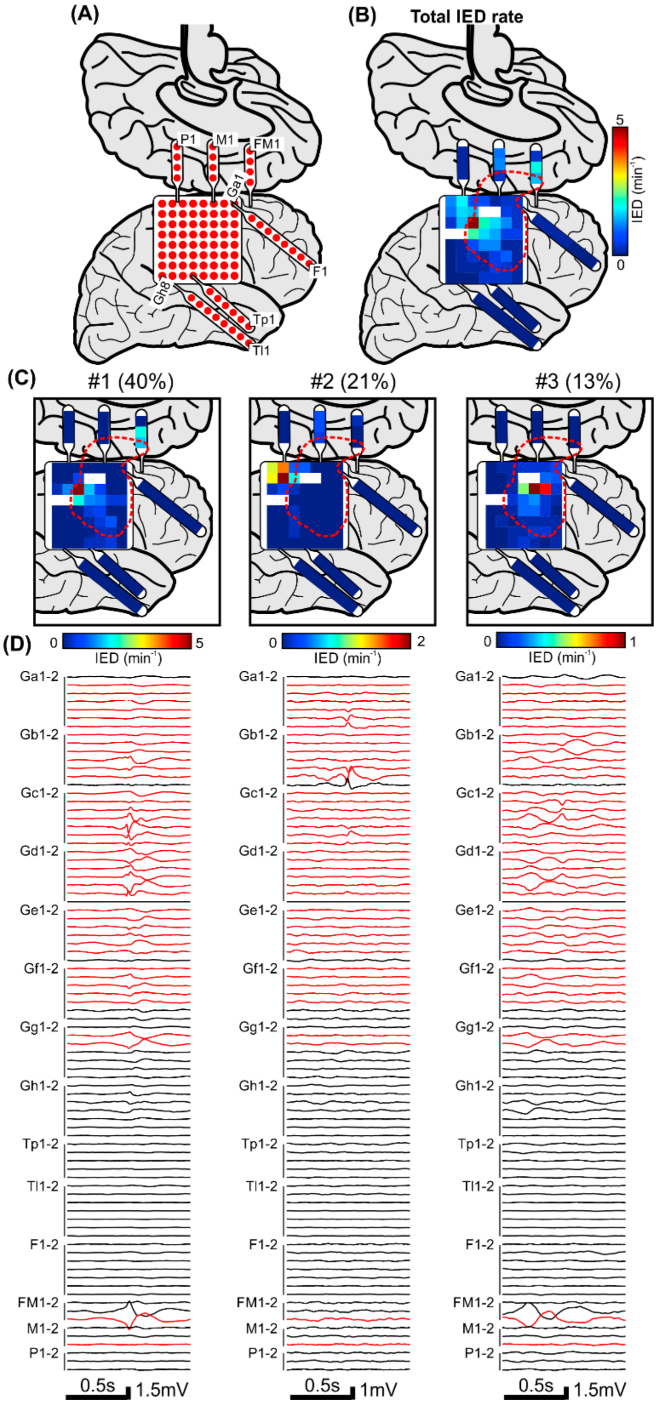

Supplementary Figure 8. Analysis of the spatial profile of IEDs (patient P7). (A) The schematics of electrode placement. (B) Spatial profile of all detected IED distributed above central region. (C) Three major sub-regions (C), their corresponding activity and average waveforms (D). The most active sub-region #1 and sub-region #3 was resected (red lines). The patient's outcome is Engel I.

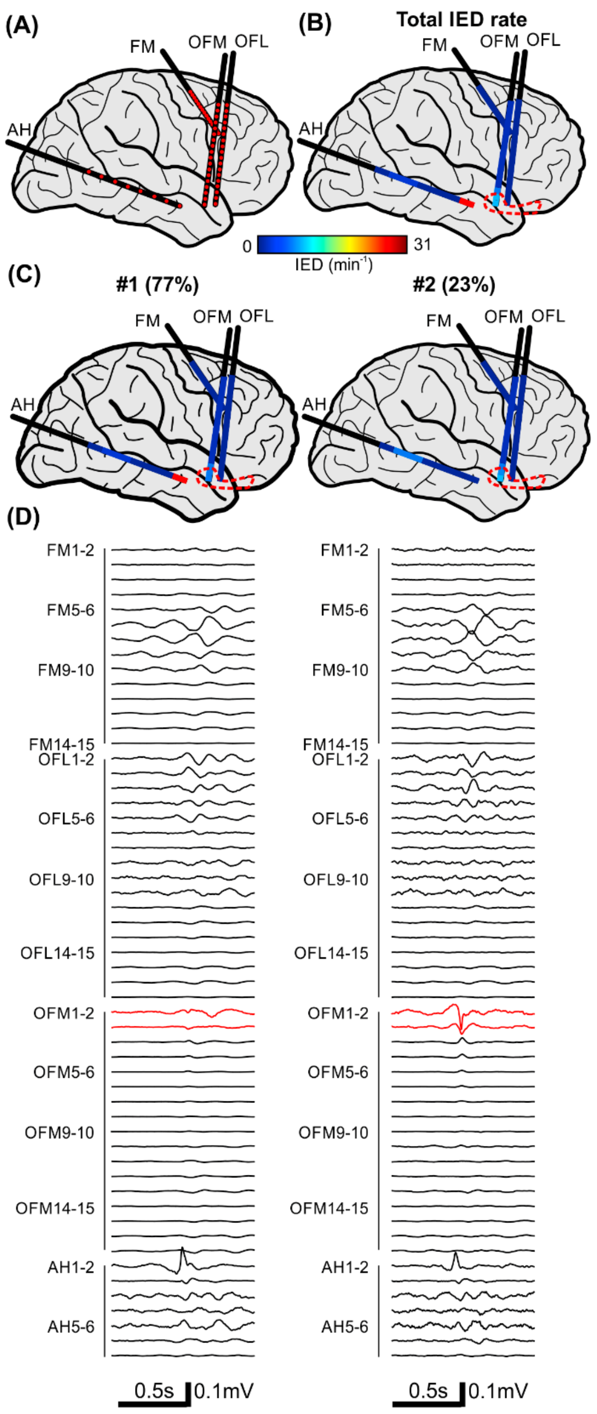

Supplementary Figure 9. Analysis of the spatial profile of IEDs (patient P8). (A) The location of intracerebral electrodes. (B) Spatial profile of all detected IEDs demonstrates the maxima distribution of IED activity orbitofrontal (OFM) electrode and electrodes located in amygdalo-hippocampal complex (AH). (C) Two sub-regions were identified. (D) Average waveforms for each sub-region. The most active sub-region #1 was localized outside the seizure onset zone, and it was not included in the resection (red line). The patient's outcome is Engel II.

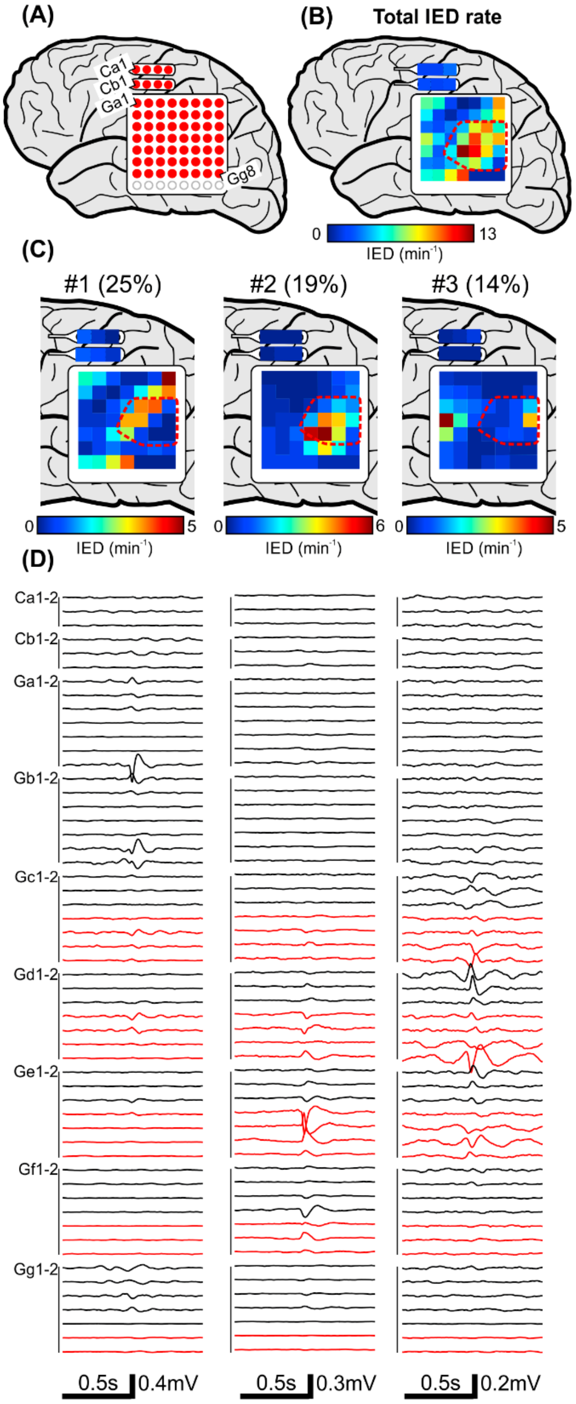

Supplementary Figure 10. Analysis of the spatial profile of IEDs (patient P9). (A) The schematics of electrode placement. (B) Spatial profile of all detected IEDs which display large spatial distribution over a temporoparietal area. (C) Three main identified sub-regions, their corresponding activity and average waveforms (D). The most active sub-region #1 was only partially resected (red lines). The most active channels Ga7-8 of the sub-region #1 was not resected. The patient's outcome is Engel III.

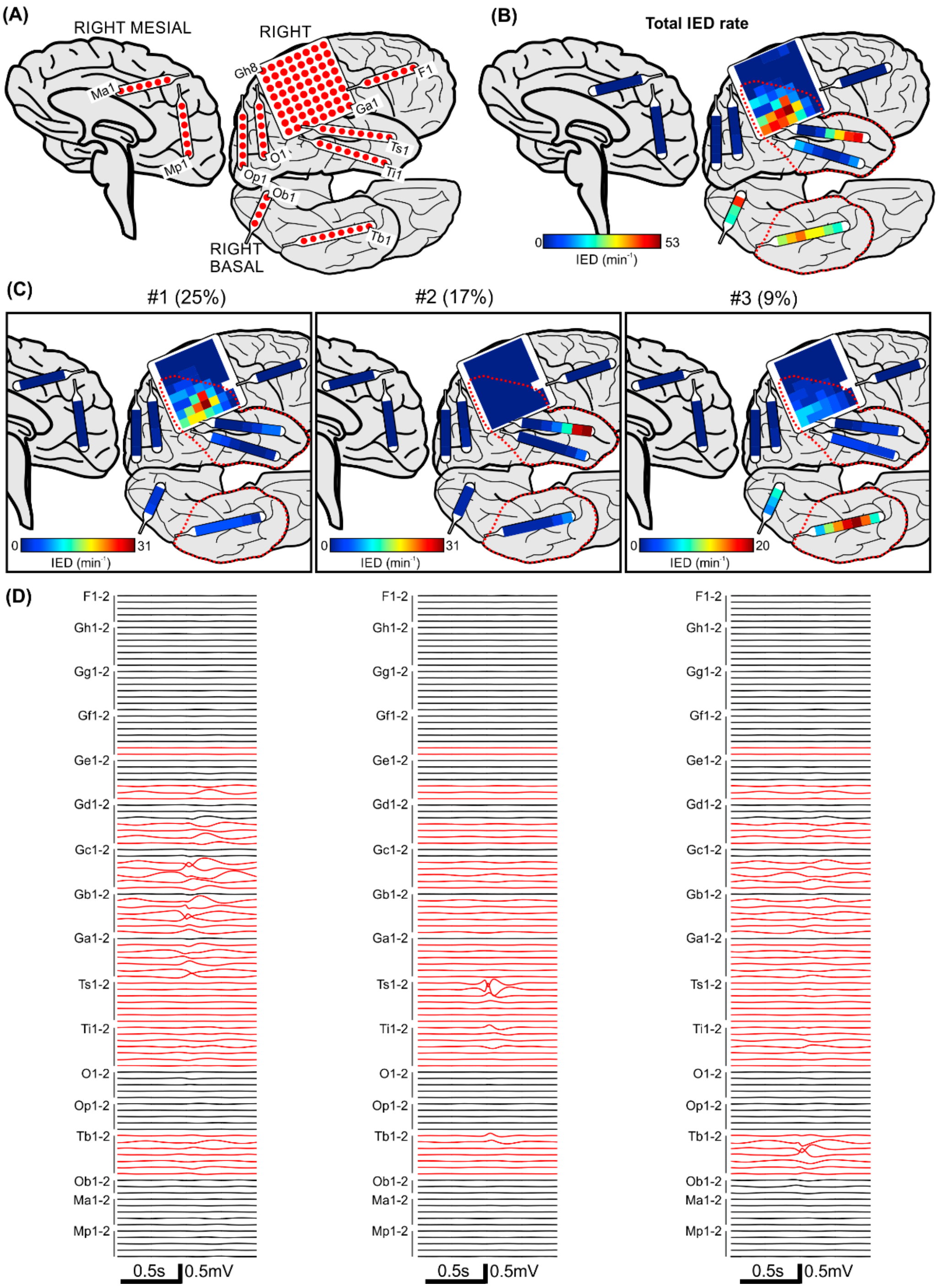

Supplementary Figure 11. Analysis of the spatial profile of IEDs (patient P10). (A) The schematics of electrode implantation. (B) Spatial profile of all detected IED which are distributed over a temporoparietooccipital area. (C) Three identified sub-regions, their corresponding activity and average waveforms (D). The majority of sub-regions were included in the resection (red lines). The patient’s outcome is Engel IV.

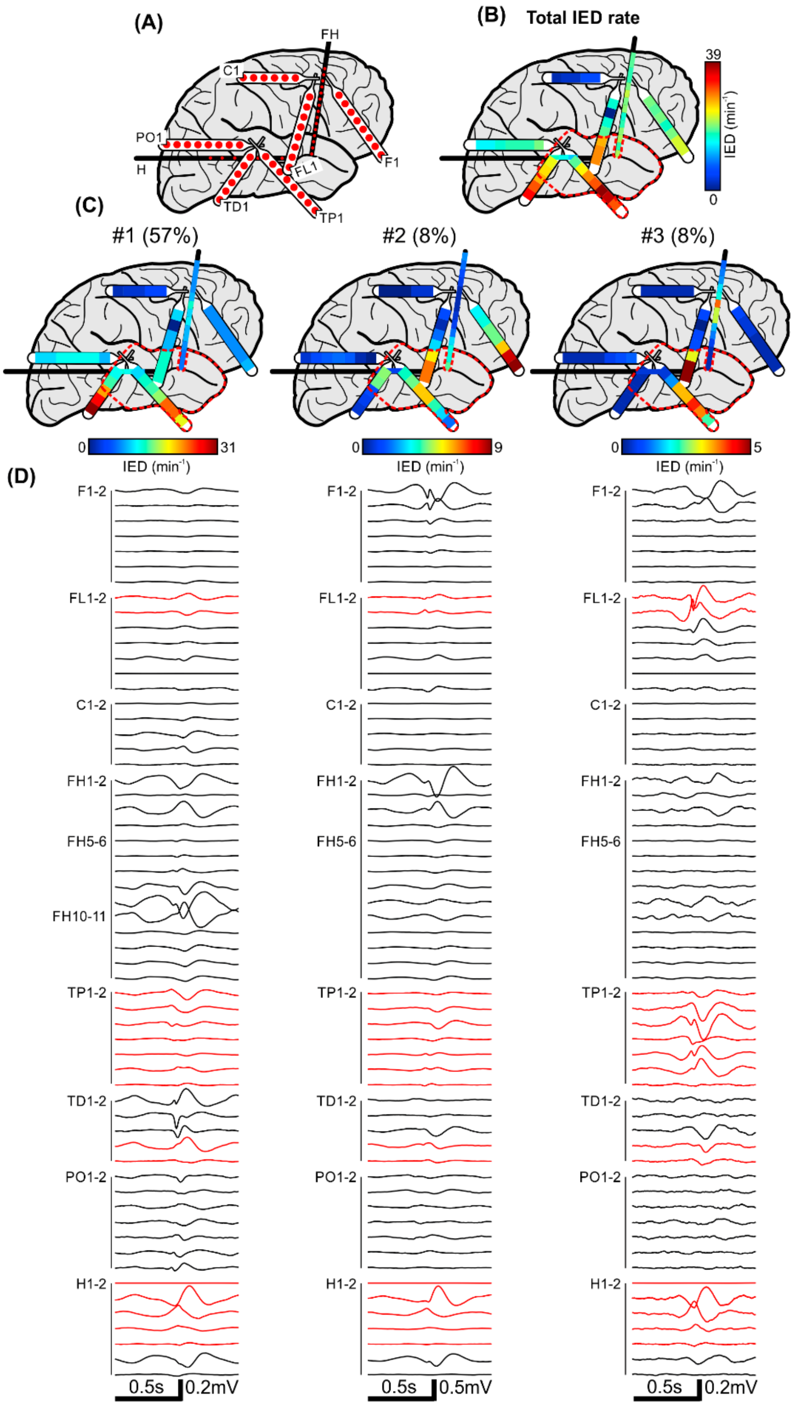

Supplementary Figure 12. Analysis of the spatial profile of IEDs (patient P11). (A) Positions of implanted electrodes. (B). Spatially extensive distribution of detected IEDs in the right hemisphere. The maximum of the IED occurrence seems to be located at the base of the right temporal pole (C) Three major sub-regions and average their IED waveforms (D). The analysis revealed that the dominant sub-region #1 was in a located in a temporooccipital area outside the resection (red line). The patient's outcome is Engel IV.

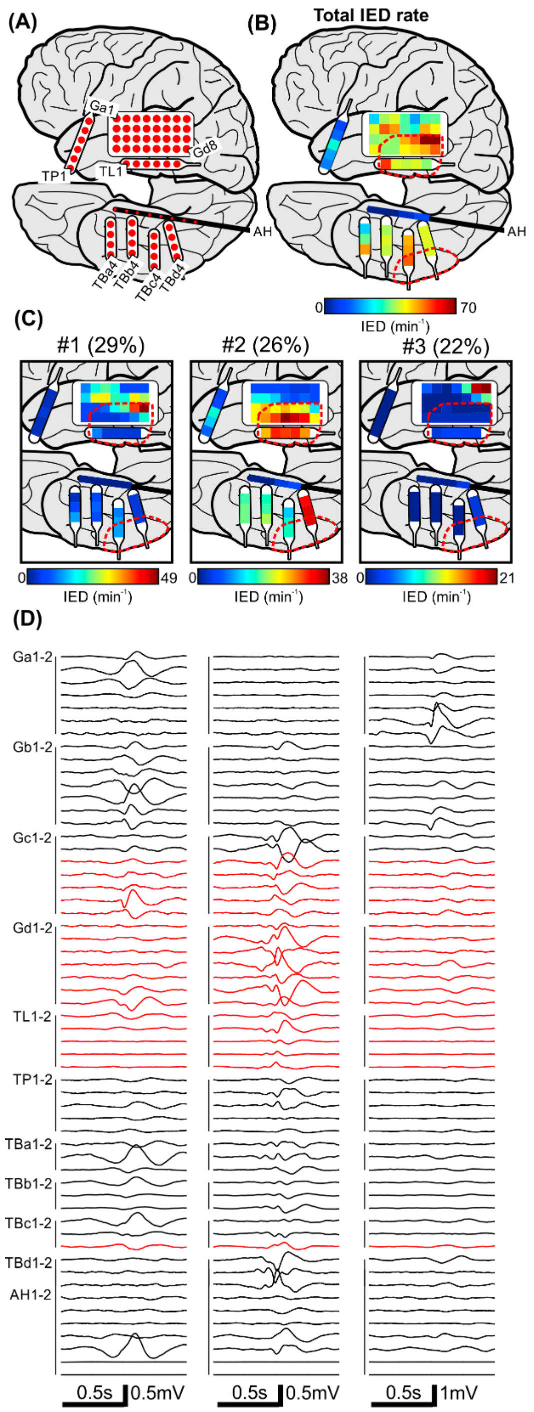

Supplementary Figure 13. Analysis of the spatial profile of IEDs (patient P12). (A) The schematics of the electrodes. (B) The total IED activity which is distributed over the temporal lobe. (C) Three major sub-regions, their corresponding activity and average waveforms (D) are shown. Sub-regions #1 and #2 were in included in resection (red line). Sub-region #3 located in a temporoparietal region generated a substantial amount of IED activity. This sub-region wasn't included in the resection. The patient's outcome is Engel IV.

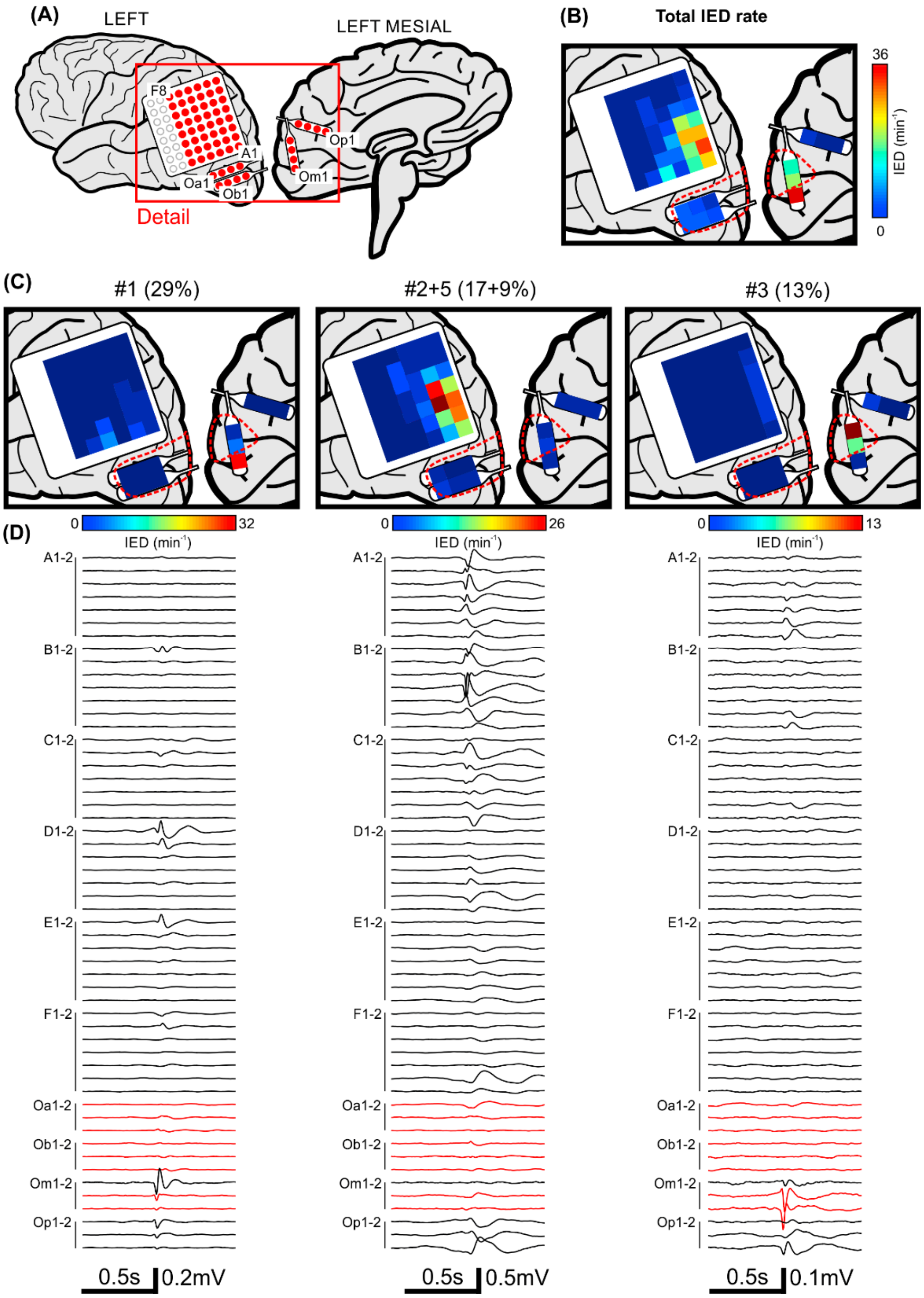

Supplementary Figure 14. Analysis of the spatial profile of IEDs (patient P13). (A) The schematics of electrode implantation. (B) Spatial profile of all detected IEDs distributed over a parietooccipital area. (C) Three main sub-regions with their corresponding activity and average waveforms (D). Sub-regions #1 and #2 were not resected (red line). The patient's outcome is Engel IV.

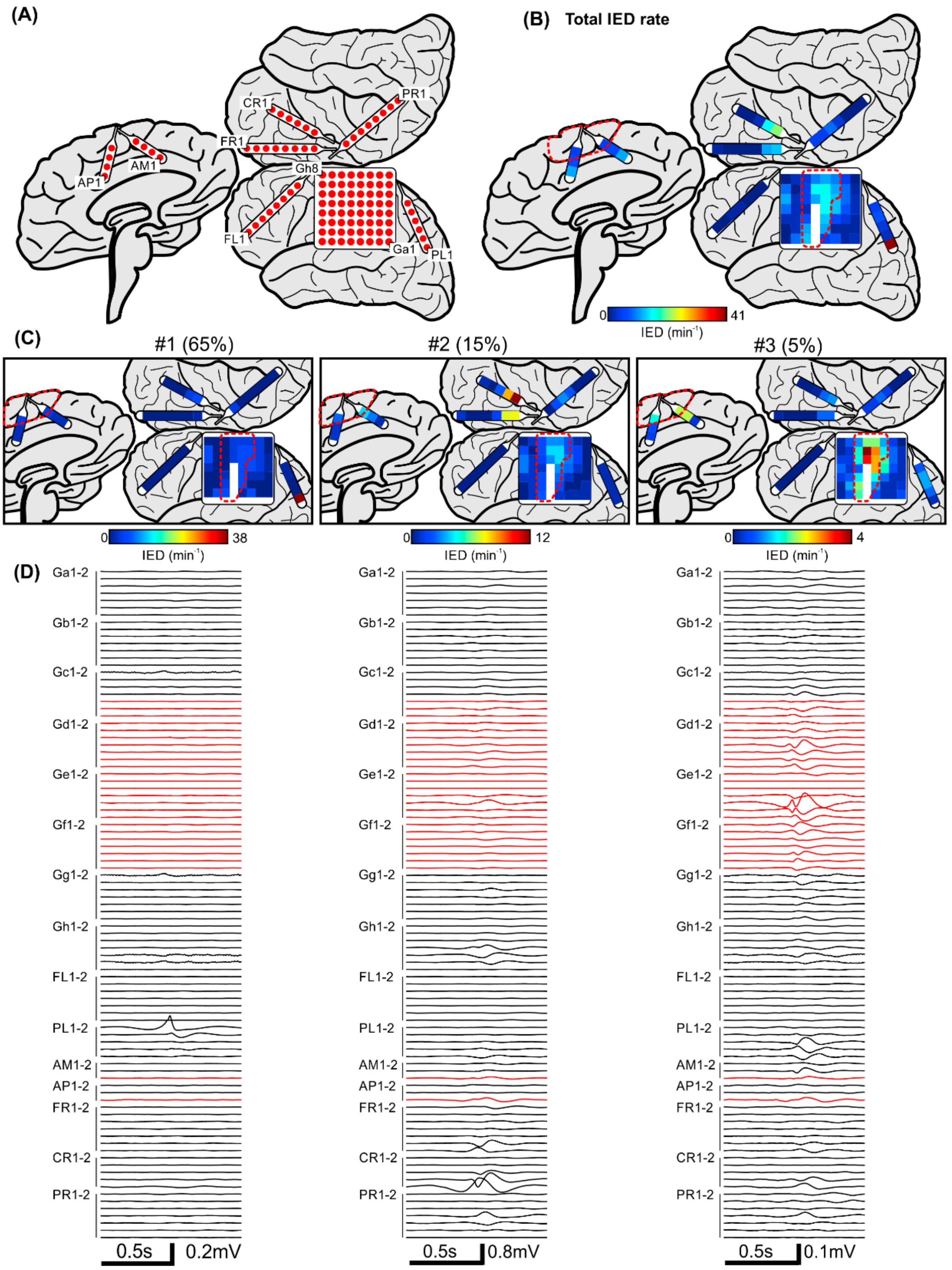

Supplementary Figure 15. Analysis of the spatial profile of IEDs (patient P14). (A) The electrode placement. (B). Spatial distribution of all detected IEDs. (C)Three identified sub-regions, their corresponding activity and average waveforms (D). The most active sub-regions #1 and #2 were not in resection (red line). The patient's outcome is Engel IV.
